# Neoadjuvant chemoradiotherapy or radiotherapy in patients aged 75 years or older with locally advanced rectal cancer

**DOI:** 10.7150/jca.41950

**Published:** 2020-03-15

**Authors:** Xiaoliang Liu, Junjie Wang, Ke Hu, Fuquan Zhang, Xiaorong Hou, Yi Xiao, Xin Lian, Shuai Sun, Zhikai Liu, Junfang Yan, Zheng Miao

**Affiliations:** 1 Department of Radiation Oncology, Peking Union Medical College Hospital, Chinese Academy of Medical Sciences & Peking Union Medical College, Beijing, the People's Republic of China.; 2 Department of Gynecological Oncology, Qingdao Center Hospital, Qingdao, Shandong, the People's Republic of China.; 3 Department of Surgery, Peking Union Medical College Hospital, Chinese Academy of Medical Sciences & Peking Union Medical College, Beijing, the People's Republic of China.

**Keywords:** rectal cancer, elderly, neoadjuvant, radiotherapy, chemotherapy

## Abstract

**Background**: To evaluate the efficacy and treatment related morbidity of neoadjuvant chemoradiotherapy or radiotherapy in elderly patients (aged 75 years or older) with locally advanced rectal cancer (LARC).

**Methods**: We reviewed clinical records of elderly patients with LARC treated with neoadjuvant chemoradiotherapy or radiotherapy between January 2008 and June 2017 at our institute. A dose of 45-50Gy in 25 fractions was delivered to pelvis. The primary tumor received a dose of 55Gy concomitantly for patients receiving intensity modulated radiotherapy (IMRT). The concurrent chemotherapy included capecitabine alone and capecitabine plus oxaliplatin (Xelox). Surgery was performed for suitable patients at least 6 weeks after neoadjuvant treatment. Overall survival (OS), disease specific survival (DSS), disease free survival (DFS) and local control (LC) were calculated with Kaplan-Meier method.

**Results**: A total of 85 patients were enrolled in this study, the median age was 80 years old (range: 75-90 years). After neoadjuvant treatment, surgery was performed in 56 patients (65.9%). Downstaging rate was 85.7% (48/56) with T downstaging in 35 patients (62.5%) and N downstaging in 36 patients (64.3%). Twelve patients (21.4%) obtained pathological complete response (pCR). The incidence of grade 3 or greater acute hematological, gastrointestinal and genitourinary toxicities were 10.7%, 5.2% and 1.8%, respectively. Seven patients (12.5%) experienced postoperative complications. The median follow-up duration was 35.7 months (range: 4.3-100.3 months), The 3-year and 5-year OS, DSS, DFS, LC were 68.9% and 47.2%, 75.8% and 60.4%, 68.2% and 56.1%, 83.9% and 78.3%, respectively.

**Conclusion**: In patients aged 75 years or older with LARC, neoadjuvant chemoradiotherapy followed by surgery was well tolerated with promising survival outcomes, which should be strongly suggested if medically suitable.

## Background

Surgery, radiotherapy and chemotherapy are the primary treatment methods for patients with locally advanced rectal cancer (LARC). Compared with other modalities, neoadjuvant chemoradiotherapy followed by total mesorectal excision (TME) achieved better survival outcomes and fewer treatment related toxicities 1-5. Therefore, this method has been identified as the standard treatment for patients with LARC.

As life expectancy increases, more and more elderly people suffer from rectal cancer 6. With the increasing age, patients are more prone to suffer comorbidities and treatment related morbidities 7-10. In a retrospective study 11, patients older than 75 years had worsen comorbidity status and more medical complications than those younger than 75 years. A report from ACCOR 12/PRODIGE 2 phase III trial revealed that elderly patients (≥70 years) with LARC experienced more G3/4 toxicities than younger patients (<70 years) when performing preoperative chemoradiotherapy (25.6% vs 15.8%, P=0.01) 12. Another study compared surgery related morbidities between older and younger patients 13, the results showed greater intraoperative blood loss and a higher surgical conversion rate in older patients (≥70 years). All these negative effects may compromise the efficacy of treatment.

To achieve promising results, many clinical trials usually exclude elderly patients from the study group. In the study of FFCD9203 2, all eligible patients were limited to younger than 75 years and with WHO performance status of 0 or 1. In a phase III randomized trial comparing two neoadjuvant therapies for LARC 14, patients older than 75 years were excluded from the study. The CAO/ARO/ AIO-94 randomized phase III trial also only involved patients younger than 75 years 3. These milestone studies all underrepresented elderly patients with LARC. The treatment options for elderly patients with LARC are mainly extrapolated from results concluded with relative younger patients. The best treatment choice for this group of patients is still unclear.

This retrospective study was designed to evaluate the tolerance and efficacy of neoadjuvant chemoradiotherapy or radiotherapy in elderly patients with LARC.

## Methods

### Patients selection

After receiving approval from the Institutional Review Board (IRB) of Peking Union Medical College Hospital, we reviewed the clinical records of patients with LARC treated with neoadjuvant chemoradiotherapy or radiotherapy from January 2008 to June 2017 at our institute. The inclusion criteria were as follows: histologically proven rectal cancer; with age ≥75 years; with locally advanced stage (cT3+ or cN+); scheduled to receive neoadjuvant chemoradiotherapy or radiotherapy. The pretreatment evaluation included complete history and physical examinations, digital rectal examination (DRE), complete blood counts and biochemical analysis, tumor markers (CEA and CA199), colonoscopy, thoracic and abdominal computed tomography (CT), rectal magnetic resonance imaging (MRI) and transrectal ultrasound. The positron emission tomography/CT (PET/CT) were not routinely used.

### Radiotherapy

As described in our previous study 15, all eligible patients received pelvic irradiation with 3 dimensional conformal radiotherapy (3D-CRT) or intensity modulated radiotherapy (IMRT). Clinical target volume (CTV) included the complete mesorectum and pelvic lymph node region (presacral, internal iliac and obturator lymph node). Gross tumor volume (GTV) consisted of the primary tumor (GTV-T) and the positive pelvic lymph nodes (GTV-N). Positive lymph node was defined as short diameter ≥1 cm on MRI or confirmed by PET/CT. Planning clinical target volume (PCTV) was defined as CTV plus a 6-10 mm margin. A margin of 5mm was added to GTV to form the planning gross tumor volume (PGTV).

A dose of 45-50Gy in 25 fractions was delivered to at least 95% of the PCTV. For patients receiving IMRT, at least 95% of the PGTV was escalated to 55Gy in 25 fractions with a simultaneously integrated boost (SIB) technique.

All patients treated with IMRT received image guidance at our institute. Patients who received volumetric modulated arc radiotherapy (VMAT) underwent cone beam computed tomography (CBCT) weekly. Daily on-board megavoltage CT (MVCT) was performed for patients receiving helical tomotherapy (HT).

### Chemotherapy

Concurrent chemotherapy was scheduled to perform for eligible patients. Regimens included oral capecitabine (825mg/m^2^, d1-14) with or without oxaliplatin (130mg/m^2^, d1, Xelox). All patients were scheduled for two cycles of concurrent chemotherapy. Adjuvant chemotherapy was based on the postoperative pathological examinations and patients' conditions.

### Surgery

Before surgery, rectal MRI and/or transrectal ultrasound were performed for patients to reevaluate the stage and possibility of resection. Whether to perform the surgery and the surgery modalities depended on patients' conditions, preferences and the attending surgeons. Surgery was generally conducted six to eight weeks after the neoadjuvant treatment. Patients with low rectal cancer who received sphincter-preservation TME receiving prophylactic ileostomy. Transanal endoscopic microsurgery (TEM) was an alternative for some patients with clinical complete response (cCR), very low rectal cancer (<3cm from the anal verge) and a strong desire for sphincter-preserving. “Watch-and-wait” approach was also a choice for patients with cCR.

### Follow-up and toxicity evaluation

After surgery, patients received regular follow-up every three months in the first two years, then every six months during the next 3-5 years and once a year thereafter. The routine follow-up examinations included DRE, complete blood counts, liver and renal functions, carcinoembryonic antigen (CEA) and carbohydrate antigen 19-9 (CA19-9), colonoscopy, thoracic and abdominal CT, pelvic MRI. PET/CT was not recommended unless suspect of disease relapse. Acute neoadjuvant chemoradiotherapy related toxicities were assessed with Common Terminology Criteria for Adverse Events, version 3.0 (CTCAE 3.0).

### Statistical analyses

Overall survival (OS) was defined as time from the start of the treatment to the date of death or last follow-up. Disease specific survival (DSS) referred to the date from the start of treatment to the death date due to the disease or the time of the last follow-up. Disease free survival (DFS) was counted from the start of the treatment to the time of any disease relapse or the last follow-up. The time from the start of treatment to the date of local recurrence or last follow-up was defined as local control (LC). OS, DSS, DFS and LC were calculated with Kaplan-Meier methods. All statistical analyses were performed with SPSS 23.0 software (SPSS Inc, Chicago, IL, USA). A two side P value of <0.05 was defined as statistically significant.

## Results

### Patients' characteristics

From January 2008 to June 2017, eighty-five elderly patients with LARC were scheduled for neoadjuvant chemoradiotherapy or radiotherapy at our institute. The median age was 80 years old (range: 75-85 years). All patients were diagnosed with rectal adenocarcinoma, with 42 patients (49.4%) in the low rectum and 43 patients (50.6%) in the middle rectum. Seventy-nine patients (92.9%) had clinical T3 or T4 stage. Positive lymph nodes were involved in 61 patients (71.8%). The detailed information of patients' characteristics is listed in Table 1.

### Neoadjuvant treatment

All patients completed the radiotherapy procedure with 3D-CRT in seven patients (8.2%), VMAT in 65 patients (76.5%) and HT in 13 patients (15.3%). The median radiotherapy duration was 35 days (range: 30-50 days). Four patients (5.9%) had prolonged radiotherapy because of grade 3 or 4 hematological toxicity. One patient suffered stenocardia when he finished 11 fractions of radiotherapy, and he received symptomatic treatment and complete cardiac examination in another specialized hospital. This patient completed the rest fractions after recovery. However, detailed treatment information about his heart disease was not available due to the retrospective nature and information sharing reason.

Twelve patients (14.1%) didn't receive concurrent chemotherapy due to poor healthy conditions or patients' refusal. Fifty-four (63.5%) and nineteen patients (22.4%) underwent capecitabine alone and Xelox, respectively.

### Surgery

After neoadjuvant treatment, sixty-two patients received reevaluation of the tumor stage. Clinical complete response (cCR) occurred in 11 patients (17.7%). Among them, seven patients received a “watch and wait” approach instead of definitive surgery, two patients had TME surgery, whereas the other two patients underwent TEM surgery.

A total of 56 patients (65.9%) received surgery after neoadjuvant treatment. Forty-eight patients underwent TME surgery, including Dixon in 35 patients (72.9%), Miles in 10 patients (20.8%). Hartmann in two patients (4.2%), and Parks in one patient (2.1%). While TEM surgery was performed in eight patients. For patients who had TME, laparoscopic surgery was performed in 41 patients (85.4%), and open surgery was only in seven patients (14.6%). Sphincter-preserving surgery was performed in 44 patients (78.6%).

Twenty-nine patients (34.1%) didn't undergo surgery for several reasons. Seven patients with cCR received a “watch-and-wait” strategy, two patients who were not suitable for surgery had a primary tumor boost irradiation of 10Gy in 5 fractions, the other 20 patients refused surgery due to personal reasons.

### Treatment related toxicities and complications

Acute toxicity was defined as toxicities occurred during treatment and within three months after treatment. A total of 56 patients were available for scoring neoadjuvant treatment related toxicities, in which nine patients (16.8%) suffered grade 3 or greater toxicities. The incidence of acute grade 3 or greater acute hematologic, gastrointestinal and genitourinary toxicity were 10.7%, 5.2% and 1.8%, respectively. Seven patients (12.5%) experienced postoperative complications including anastomotic fistula, ileus and abdominal infection (Table 2). There was no treatment related death in our study.

### Pathological evaluation

After neoadjuvant treatment and surgery, pathological examination identified forty-eight patients (48/56, 85.7%) with pathological downstaging including T downstaging in 35 patients (62.5%) and N downstaging in 36 patients (64.3%). Pathological complete response (pCR) occurred in 12 patients, which accounted for 21.4% of patients receiving surgery. Of the 39 patients with N+ stage before treatment, negative lymph node was observed in 33 patients (84.6%).

### Survivals and patterns of failure

**Figure 1:**
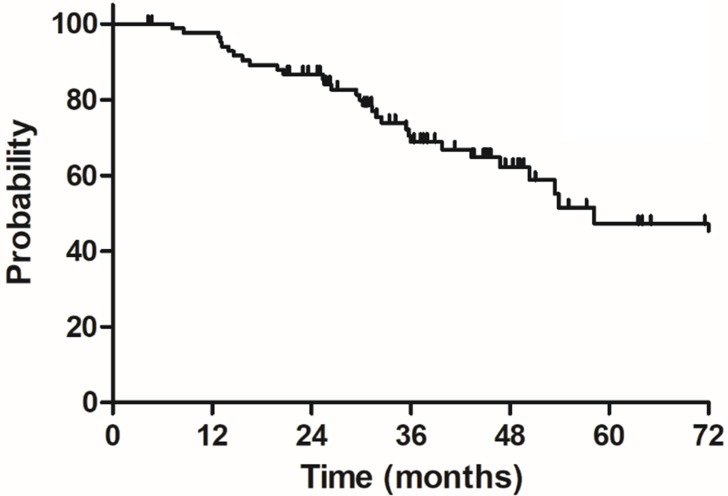
Overall survival (OS) for 85 elderly patients with LARC.

**Figure 2:**
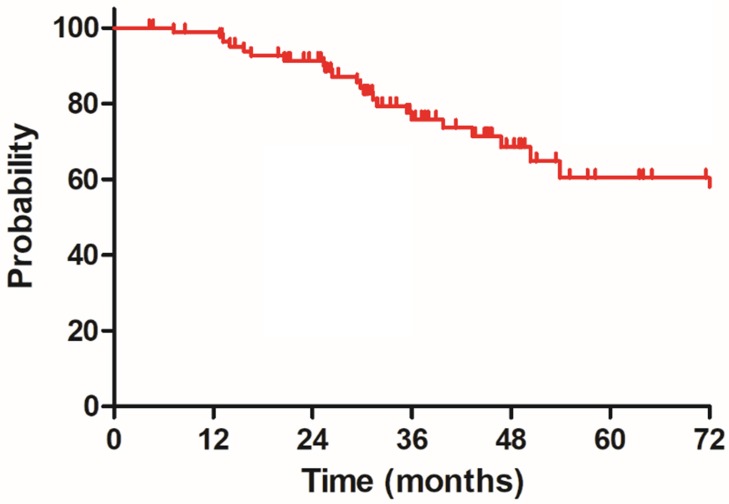
Disease specific survival (DSS) for 85 patients with LARC.

**Figure 3:**
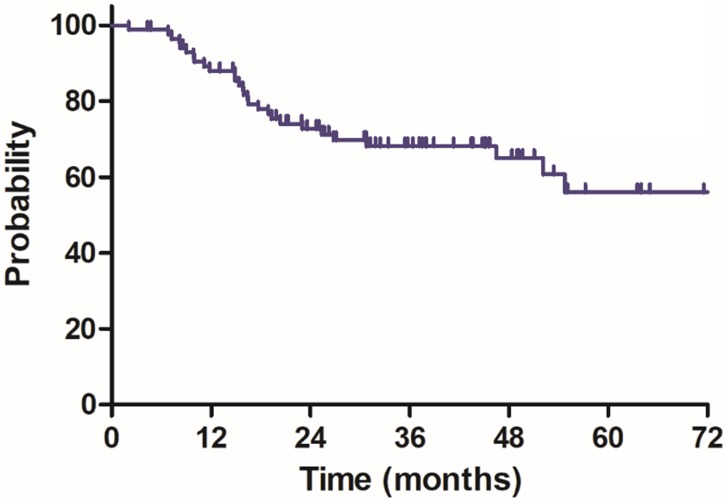
Disease free survival (DFS) for 85 patients with LARC.

**Figure 4:**
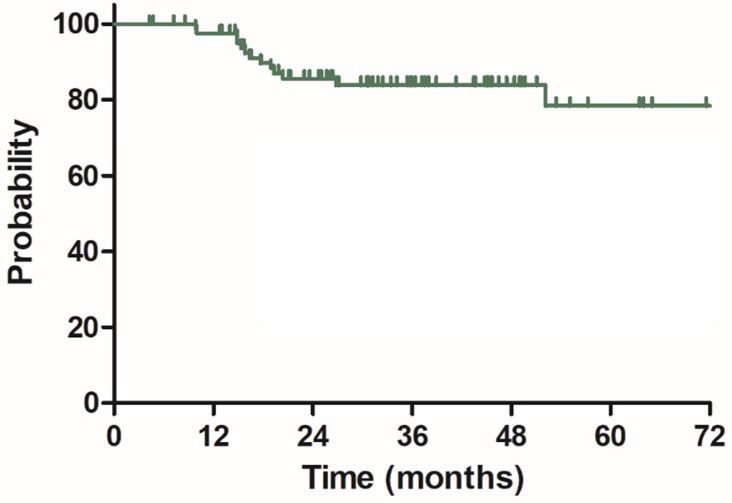
Local control (LC) for 85 patients with LARC.

The median follow-up duration was 35.7 months (range: 4.3-100.3 months), twenty-three patients died of rectal cancer, whereas eight patients died due to other comorbidities during the follow-up. The 3-year OS, DSS, DFS and LC were 68.9%, 75.8%, 68.2% and 83.9%, respectively, the estimated 5-year OS, DSS, DFS and LC were 47.2%, 60.4%, 56.1% and 78.3%, respectively (Figure 1-4).

By the end of last follow-up, twenty-eight patients (32.9%) suffered disease recurrence including ten patients (11.8%) with local relapse only, fifteen patients (17.6%) with distant metastasis only and three patients (3.5%) with both local relapse and distant metastasis. Lung and liver were the most prevalent organs for distant metastasis.

## Discussion

The best treatment option for elderly patients with LARC remained controversial due to lacking in prospective clinical data 16, 17. Though neoadjuvant chemoradiotherapy combined with TME surgery is the current standard treatment, many elderly patients can't finish the whole treatment procedure because of comorbidities, treatment related morbidities and other personal reasons 7, 9, 16, 18-20. Our study enrolled 85 patients at least 75 years with LARC. Fifty-six patients (65.9%) completed the scheduled neoadjuvant treatment and surgery procedure. We achieved promising survival outcomes with acceptable treatment related toxicities and complications.

Many studies have reported the incidence of neoadjuvant chemoradiotherapy related toxicity in elderly patients with LARC 12, 19-22. Tougeron D, et al 20 retrospectively evaluated safety of chemoradiotherapy in 125 patients with LARC over 70 years, about 15% of enrolled patients developed G3+ adverse events. In the study of ACCOR 12/PRODICE 2 phase III trial 12, the incidence of severe grade 3/4 preoperative chemoradiotherapy related toxicity in elderly patients (≥70 years) was 25.6%, while it was only 15.8% in younger patients (<70 years). A case-matched control study from Korea also showed higher rate of G3+ acute hematologic toxicity in elderly patients (≥70 years), compared with that in patients younger than 70 years (16.1% vs 9.0%) 21. In our study, nine patients (16.1%) developed G3/4 neoadjuvant treatment related toxicities, which was comparable with the above studies. Compared with our previous report 15 which included patients with a median age of 59 years (range: 50-67 years), patients in the present study also experienced more G3+ toxicities. Based on these findings, the incidence of neoadjuvant treatment related toxicity in elderly patients with LARC is indeed higher than that in younger patients, but it is quite acceptable. On the other hand, all patients in our study completed the radiotherapy procedure and only five patients experienced prolonged radiotherapy. This also indicated that neoadjuvant treatment is well tolerated in elderly patients with LARC. Surgery morbidity and mortality are another concerns for clinicians when performing surgery in elderly patients 16. It was reported that the mortality rate after TME surgery was at least 2-5%, and even higher in older patients 16. With the improvement of surgical and anesthesiological techniques, postoperative nutritional support and physical activity interventions, postoperative morbidity and mortality in most elderly patients are no longer different from their younger counterparts 16. In a prospective cohort study, the rates of postoperative complications for elderly patients and younger counterparts were 38.5% and 34.7% 10. As for the present study, most TME surgeries were performed with laparoscopic technique (85.4%), the postoperative morbidity rate was only 12.5%.

The rate of pCR is an important factor for assessing the effect of neoadjuvant chemoradiotherapy and it is also related with improved survival outcomes 15. The pCR rate is significantly influenced by the escalated dose to primary tumor. When receiving ≥60 Gy irradiation, the pooled pCR rate was 20.4% in a mata-analysis 23. A prospective study from China recruited 63 patients with LARC, patients received IMRT with pelvic to 41.8Gy in 22 fractions and primary tumor to 50.6Gy simultaneously. The pCR rate was as high as 31.0% 24. Zhu, et al also investigated the effect of concomitant boost irradiation on pCR rate in a phase II study 25. The prescribed doses to the pelvic area and primary tumor were 50Gy in 25 fractions and 55Gy in 25 fractions, respectively. A total of 18 in 78 patients (23.7%) obtained pCR after surgery. However, when patients were treated without primary tumor dose escalation, the reported pCR rates only varied from 11.4% to 19.2 2, 26-28. Regarding elderly patients with LARC, Jacobs L, et al retrospectively reviewed 42 patients aged ≥70 years receiving neoadjuvant chemoradiotherapy, the irradiation dose to pelvic region was 50Gy in 25 fractions without primary tumor boost, pCR was merely observed in 3 patients (7.5%). In the present study, 91.8% of eligible patients received IMRT with boost irradiation to primary tumor of 55Gy in 25 fractions. The pCR rate was 21.4%, which was consistent with the previous meta-analysis 23. Another propensity-score matching analysis 21 reported that the pCR rate wasn't significantly different between elderly and younger patients (14.8% vs 17.1%, P=0.433). Obviously, elderly patients can also get benefit from dose escalation radiotherapy as their younger counterparts do.

There is another key point that should not be ignored. With increasing age, more patients would die from other comorbidities 7, such as cardiovascular, nervous system and pulmonary diseases. Eight patients died of comorbidities instead of rectal cancer, accounting for 25.8% of all deceased in our study. Considering this, DSS, DFS and LC are more suitable for evaluating the efficacy of treatment in elderly patients with LARC. Our study reported that the 3-year and estimated 5-year DSS, DFS and LC for patients receiving multimodal therapy were 75.8% and 60.4%, 68.2% and 56.1%, 83.9% and 78.3%, respectively. These results were quite comparable to the previous studies regarding elderly patients 12, 20-22, 29. Notably, the oncologic outcomes of our study were not inferior than younger patients in other studies 2, 15, 21. In the study of FFCD 9203 2, the reported 5-year progression free survival (PFS) and local recurrence rate (LRR) were 59.4% and 8.1%. Recently, Sung SY, et al 21 conducted a propensity- score matching study to compare oncologic outcome and morbidity between elderly and younger patients after neoadjuvant chemoradiotherapy and TME surgery. Though the rates of acute hematologic toxicity and late complications were higher in elderly patients, no significant difference was observed regarding 5-year recurrence free survival (RFS) between elderly and younger patients (65.5% vs 67.7%, P=0.483). Thus, age may not be a determining factor when clinicians develop treatment strategies for elderly patients with LARC.

Watch-and-watch policy is an alternative for patients with cCR after neoadjuvant treatment, especially for elderly or frail patients 30-32. With this approach, patients can avoid the inconvenience of colostomy and other surgery complications without the expense of survival, which may further led to a good quality of life 33. A meta-analysis 31 enrolled 23 studies including 867 patients with respect to watch-and-wait strategy and surgery for patients with LARC. The pooled 2-year local regrowth was 15.7%, and most patients (95.4%) received salvage therapies. No significant difference was observed regarding non-regrowth recurrence, CSS, DFS and OS between patients with cCR after neoadjuvant treatment managed with watch-and-wait approach and surgery. Eleven patients obtained cCR after chemoradiotherapy in our study. Watch-and-wait approach was performed in seven patients. By the end of follow-up, only one patient suffered regrowth recurrence. The number of patients with cCR in present study was too small to draw meaningful conclusions, but to some extent, it showed promise to implement this approach in elderly patients with LARC.

Elderly patients were defined as patients older than 70 years in most of the previous studies 9, 12, 18, 21, 22. However, rectal cancer mainly affects elderly people with a peak incidence of over 80 years 6. When people reach their 80th year, there is still life expectancy of nearly 10 years for both men and women 20. From this prospective, our study including patients 75 years or older might be more representable for geratic patients in rectal cancer. Limitations still exist in our study. The major limitation must be the retrospective nature, some patients' medical records were incomplete which might compromise our conclusions. Another shortage is the relatively small number of our study group. Though we included patients treated ten years ago (2008), few elderly patients with LARC underwent neoadjuvant treatment before 2012 at our institute.

## Conclusion

In patients aged 75 years or older with LARC, neoadjuvant chemoradiotherapy followed by surgery was well tolerated with promising survival outcomes, which should be strongly suggested if medically suitable.

## Figures and Tables

**Table 1:** Characteristics of elderly patients with locally advanced rectal cancer.

Characteristics		No. (%)
**Total**		85
**Age**		Median: 80 (range: 75-90 years)
**Gender**		
Male		61 (71.8%)
Female		24 (28.2%)
**Tumor differentiation (pretreatment)**		
Poorly		9 (10.6%)
Moderately		43 (50.6%)
Well		9 (10.6%)
Undefined		24 (28.2%)
**Tumor location**		
Low (<5cm)		42 (49.4%)
Middle (5-10cm)		43 (50.6%)
**T stage (pretreatment)**		
T1		2 (2.4%)
T2		4 (4.7%)
T3		73 (85.8%)
T4		6 (7.1%)
**N stage (pretreatment)**		
N0		24 (28.2%)
N1		39 (45.9%)
N2		22 (25.9%)
**Concurrent chemotherapy**		
None		12 (14.1%)
Capecitabine		54 (63.5%)
Xelox		19 (22.4%)
**Radiotherapy**		
3D-CRT		7 (8.2%)
VMAT		65 (76.5%)
HT		13 (15.3%)
**Surgery**		
None		29 (34.1%)
TME		48 (56.5%)
	Dixon	35 (72.9%)
	Miles	10 (20.8%)
	Hartmann	2 (4.2%)
	Parks	1 (2.1%)
TEM		8 (9.4%)

Abbreviations 3D-CRT: 3-dimensional conformal radiotherapy, VMAT: volumetric modulated arc radiotherapy, HT: helical tomotherapy, TME: total mesorecal excision, TEM: transanal endoscopic microsurgery, Xelox: capecitabine plus oxaliplatin.

**Table 2:** Treatment related toxicities and complications

nCRT toxicity (56 patients available)	Grade 3-4 No. (%)	Surgery complications (56 patients)	No. (%)
Hematologic	6 (10.7%)	Anastomotic fistula	2 (3.6%)
Gastrointestinal	3 (5.2%)	Ileus	3 (5.4%)
Genitourinary	1 (1.8%)	Infection	3 (5.4%)

Abbreviations nCRT: neoadjuvant chemoradiotherapy. Notes: Some patients suffered more than one kind of toxicity or complication.
